# Immobilized copper nanoparticles on nitrogen-rich porous activated carbon from egg white biomass: a robust hydrophilic–hydrophobic balance catalyst for click reaction

**DOI:** 10.1039/c8ra08376b

**Published:** 2018-11-19

**Authors:** Hamid Saeidian, Saleh Vahdati Khajeh, Zohreh Mirjafary, Bagher Eftekhari-Sis

**Affiliations:** Department of Science, Payame Noor University (PNU) PO Box: 19395-4697 Tehran Iran Saeidian1980@gmail.com; Department of Chemistry, Faculty of Science, University of Maragheh Golshahr, P. O. Box 55181-83111 Maragheh Iran; Department of Chemistry, Tehran Science and Research Branch, Islamic Azad University Tehran Iran

## Abstract

Biomass conversion to carbonaceous materials and their use in various applications, such as capacitors, catalyst supports, and adsorbents, have attracted considerable attention from the viewpoint of green chemistry. In this regard, chicken egg white is one of the most important biomass, which can act as an *in situ* nitrogen doping source. In this paper, nitrogen-rich porous carbon material was synthesized from egg white biomass *via* pyrolysis at 600 °C under nitrogen atmosphere, followed by chemical activation with KOH at 500 °C. The results showed that the as-synthesized porous carbon material has a high content of nitrogen and high surface area, on which nitrogen can tune the surface hydrophobicity–hydrophilicity through interaction with water molecules. Then, the copper nanoparticles were immobilized on the surface of nitrogen-rich activated carbon by a chemical reduction method. Nanocatalyst structure was characterized by elemental analysis, TEM, AFM, Raman, FT-IR, porosimetry and atomic adsorption techniques. Finally, catalytic activity was evaluated for the one-pot synthesis of triazole in aqueous medium. The nanocatalyst offers some advantages such as excellent activity, low loading of catalyst, good yields of products and short reaction times.

## Abstract 2

Nitrogen-rich porous carbon (NAC) material was synthesized from egg white biomass via pyrolysis, followed by chemical activation with KOH. Then, the copper nanoparticles were immobilized on the surface of the NAC by chemical reduction method.

## Graphical abstract

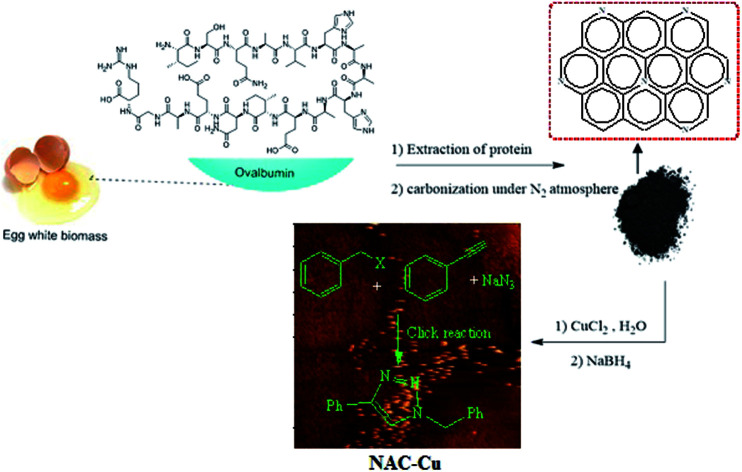

## 1. Introduction

In recent years, the chemical industry has achieved remarkably progressed in improving the quality of human life by introducing new technologies for the production of many valuable materials. Various chemical products are annually being produced with a capacity of several to hundreds of thousands of tons around the globe. During production activities in chemical industries, millions of tons of waste, such as by-products of reactions, catalysts and toxic solvents, are produced. Therefore, a combination of environmental, health and safety considerations have been examined in the production of the chemicals.^1,2^

The use of biomass materials is of high importance for producing activated carbon. Different biomasses (*i.e.*, coconut shell, wood, and agricultural waste) have been used for a long time as primary precursors in the construction of porous carbonaceous materials through thermal composition (pyrolysis). These carbonaceous materials have been extensively utilized as catalyst supports as well as in adsorption, chromatography, electrochemistry, *etc.*^3–5^ Also, due to their favorable characteristics, including high surface area, thermal and chemical stability, easy functionalization and adjustment of the surface hydrophilicity–hydrophobicity, porous activated carbon materials have attracted much attention in the field of materials science and catalysts.^6–9^ Among activated carbon materials, nitrogen-doped carbon materials have gained considerable attention because the introduction of nitrogen atoms onto the surface of activated carbon makes its surface hydrophilic.^10–12^ These compounds provide a suitable support for immobilization of various nanoparticles, and investigation of their catalytic activity as heterogeneous catalysts is a special subject in green chemistry and materials science.^13,14^ Moreover, due to the specific biological properties of triazole, it is important to provide an efficient and eco-friendly catalyst for the synthesis of these types of heterocycles.^15–19^

Due to their outstanding industrial, agrochemical, and pharmaceutical applications, 1,2,3-triazole derivatives have become one of the most important heterocycles. Alkyne-azide 1,3-dipolar cycloaddition is one of the most attractive approaches to prepare this type of heterocycles.^20^ There are many catalytic systems reported in the literature for the synthesis of 1,2,3-triazole derivatives *via* alkyne-azide 1,3-dipolar cycloaddition reaction.^21–31^ Each of the catalysts used in these reactions has its advantages and disadvantages. For example, copper(i) salts are very sensitive and will be oxidized to copper(ii) compounds when exposed to air. Therefore, the use of these catalysts under argon or nitrogen atmosphere or in the presence of sodium ascorbate is mandatory, which would lead to complicated reaction conditions. Nanocatalysts are considered as another tool in green chemistry, using which results an increase in the catalytic activity and a decrease in the amount of used catalyst are achieved due to the high surface area of these catalysts.^32–37^ Moreover, these nanoparticles (NPs) may act as a heterogeneous catalyst.

Furthermore, nanoparticle stabilization on different supports leads to more stability and ease of performance for reactions in water. The nitrogen-rich porous carbon synthesized from the biomass materials can be considered as one of the most interesting supports for stabilizing different nanoparticles through chemical reduction with NaBH_4_.^38–40^ In these supports, nitrogen can improve the immobilization of nanoparticles. The importance of heterogeneous catalysts, particularly from the economic and environmental point of view, has motivated scientists to design and introduce appropriate catalytic supports.^41–44^ Moreover, the problem of the metal particle leakage from the support's surface is an important issue that has long been studied, and many approaches have been presented for solving this problem and increasing the recyclability of the catalysts. One of these approaches is the design of catalytic supports for connecting with the metal through coordination^45,46^ or charge transfer. Then, the strong metal-support interaction prevents the leakage and accumulation of the metal particles. Due to the unique chemical and physical properties, the porous carbon materials are considered to be an appropriate option in this regard.

**Scheme 1:**
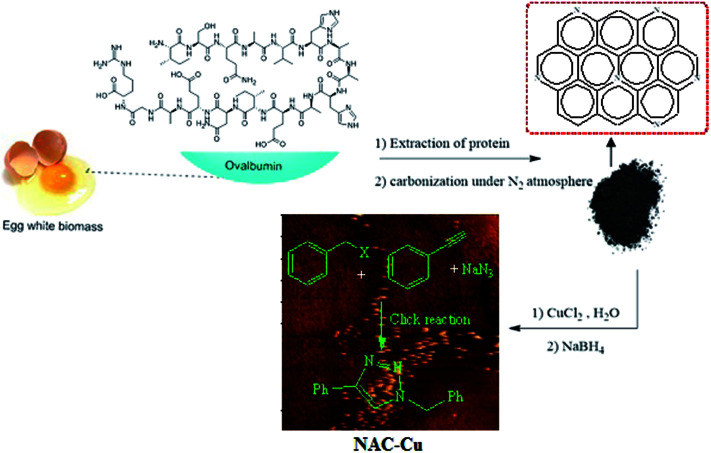
Preparation of N-rich porous carbon immobilized Cu (NAC-Cu) and its catalytic activity in the synthesis of 1,2,3-triazole.

Because of the importance of the synthesis of nitrogen-rich porous carbon supports from biomass materials and the significance of the 1,2,3-triazole synthesis, the present study aims to investigate the preparation of nitrogen-rich porous carbon supports with a large surface area from egg white as a biomass material having nitrogen bearing compounds. After the immobilization of copper nanoparticles on the nitrogen-rich porous carbon supports and characterization of the as-prepared nanocatalyst (NAC-Cu) by elemental analysis, FT-IR, Raman, TEM, AFM, AAS, BET and BJH techniques, the click reaction in the water solvent was studied in the presence of this catalyst (Scheme 1). The effects of several parameters, such as temperature, catalyst amount, and type of solvent were also investigated.

## 2. Experimental

### 2.1. Materials and instrumentation

Domestic eggs were used in this study. Sodium azide, benzyl halide derivatives, phenylacetylene and copper(ii) chloride were purchased from Aldrich Company and used without further purification.

It should be noted that before the analysis, the as-synthesized activated carbon was placed at 373 K for 12 hours. N_2_ adsorption–desorption analysis was performed for structural investigation (instrument model: Belsorp-max-BEL, Inc.). Surface area was determined using the BET calculation and pore size distribution by using the BJH method through absorption/desorption curve branch. Additional structural investigation of the as-synthesized corresponding nanocatalyst was performed using the transmission electron microscope (TEM), (Model of the PHILIPS EM2085 100 kV based in Rastak Laboratory). An atomic force microscope (AFM, DME-SPM, 95-50E) was used for obtaining AFM images. The XRD pattern of the as-synthesized nanocatalyst, obtained on Philips PW1730, was used to successfully confirm the presence of supported copper nanoparticles and identify the nanoparticle phase. The percent of graphitic structures was determined by using a Raman spectrometer (Avantes, Sensline). The FT-IR spectra were measured using the Perkin-Elmer Spectrum 65 device. Atomic adsorption measurements were recorded to confirm the immobilization of copper nanoparticles on activated carbon. The reaction progress was monitored by thin-layer chromatography, and NMR spectra of the corresponding products were obtained in the desired solvent using the Bruker 400 MHz device.

### 2.2. Preparation of activated carbon *via* pyrolysis of egg white biomass

Egg white protein was first precipitated by adding ethanol (50 mL) to a diluted egg white solution (10 mL egg white to 50 mL water). The precipitated egg white was then filtered, dried at 50 °C and pyrolyzed in a tubular furnace (600 °C for 2 h, heating rate: 5 °C min^−1^) under nitrogen atmosphere. Finally, pyrolyzed egg white (1 g) was mixed with 1 g of KOH and further activation was performed at 500 °C for 2 h under nitrogen atmosphere. In the first step, heat was applied for carbonization of biomass. In the second step, increased porosity of the obtained carbon material and activation of the nanocatalyst was achieved using KOH. The obtained carbon powder (named NAC) was washed with 100 mL HCl (2 M) overnight to remove the impurities, and rinsed 3 times with deionized (DI) water before use.

### 2.3. Immobilization of copper nanoparticles on N-doped activated carbon (NAC-Cu)

Initially, 100 mg of N-rich activated carbon (NAC) was added to 20 mL of DI water and sonicated in an ultrasonic bath for 1 h to disperse the NAC. Then, copper(ii) chloride (0.025 mmol in 2 mL DI water) was added dropwise to the mixture. After stirring for 1 h, the reaction mixture was placed in an ice-bath and 0.1 mmol of NaBH_4_, dissolved in 2 mL of DI water, was added to the mixture to reduce the concentration of copper ions and the formation of Cu NPs. The mixture was stirred at room temperature for another 1 h. The sample was centrifuged, washed with water several times and dried in vacuum for 24 h.

### 2.4. General procedure for the synthesis of 1,2,3-triazoles 3

Briefly, to a suspension of NAC-Cu (2 mol%) in H_2_O (2 mL), sodium azide (1.2 mmol, 39 mg), alkyl halide (1 mmol) and phenylacetylene (1 mmol, 110 μL) were added, and the reaction mixture was stirred at room temperature for an appropriate amount of time. The progress of the reaction was monitored by TLC. After completion of the reaction, the catalyst was recovered by filtration and washed with ethyl acetate 3 times. The catalyst was reused in a subsequent reaction without any significant loss in activity. The reaction mixture was washed with ethyl acetate (3 × 10 mL) and the organic layer was dried over anhydrous Na_2_SO_4_. Finally, solvent was evaporated and the crude product was purified by preparative TLC (eluent: petroleum ether/ethyl acetate: 4/1) to afford the desired products.

## 3. Results and discussion

**Fig. 1:**
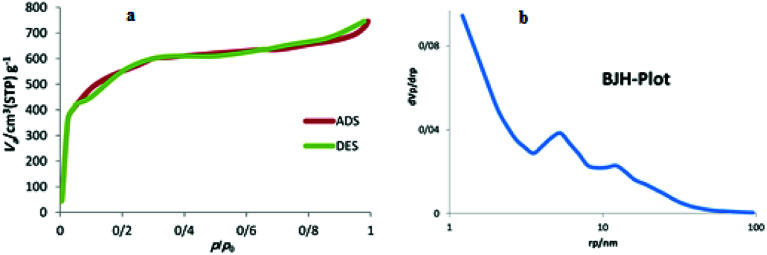
BET (a) and BJH (b) analysis of NAC-Cu.

The structure of the synthesized nanocatalyst was investigated using the porosity analysis (Brunauer–Emmett–Teller (BET) pore size analysis). Fig. 1a reveals that the as-synthesized carbon has an isotherm of type I, which is one of the characteristics of the microporous materials. The results corresponding to the distribution of the size of synthesized carbon's cavities, obtained *via* Barrett–Joyner–Halenda (BJH) analysis, indicated that the as-synthesized porous carbon support has a pore size of 1.5 nm (Fig. 1b).

Elemental analysis (CHN) results showed that amino acids in the egg white were introduced into the carbon structure, which led to nitrogenization of the final structure. The CHN analysis of NAC-Cu demonstrated that C, H and N contents of the nanocatalyst were 61.7, 0.95 and 5.61 wt%, respectively. Atomic Absorption Spectroscopy (AAS) was used to evaluate the amount of copper loaded on a carbon nanosupport, and the results showed that the loading is about 0.20 mmol g^−1^.

**Fig. 2:**
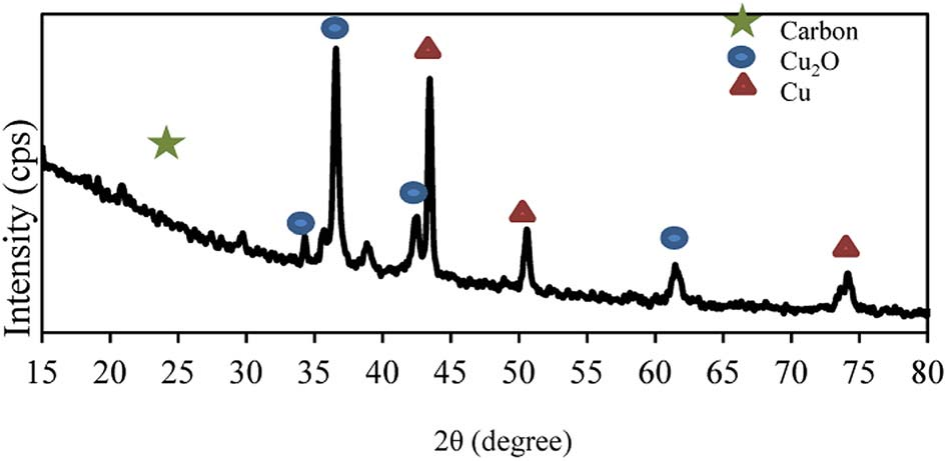
XRD pattern of NAC-Cu nanocatalyst.

Wide-angle XRD pattern is shown in Fig. 2. The broad diffraction peak of the graphite structure exists in the pattern at 2*θ* = ∼26°. Moreover, in the wide-angle XRD patterns of the NAC-Cu, the diffraction peaks at 2*θ* = ∼34°, 36°, 42°, 44°, 50°, 61° and 74° represent the crystalline forms of Cu_2_O and Cu, which formed during the preparation of NAC-Cu by the chemical reduction with NaBH_4_. The pattern also reveals that the copper particles are nanostructured, and a value of 40 and 45 nm was calculated for the Cu crystallite size using Scherrer's equation.

**Fig. 3:**
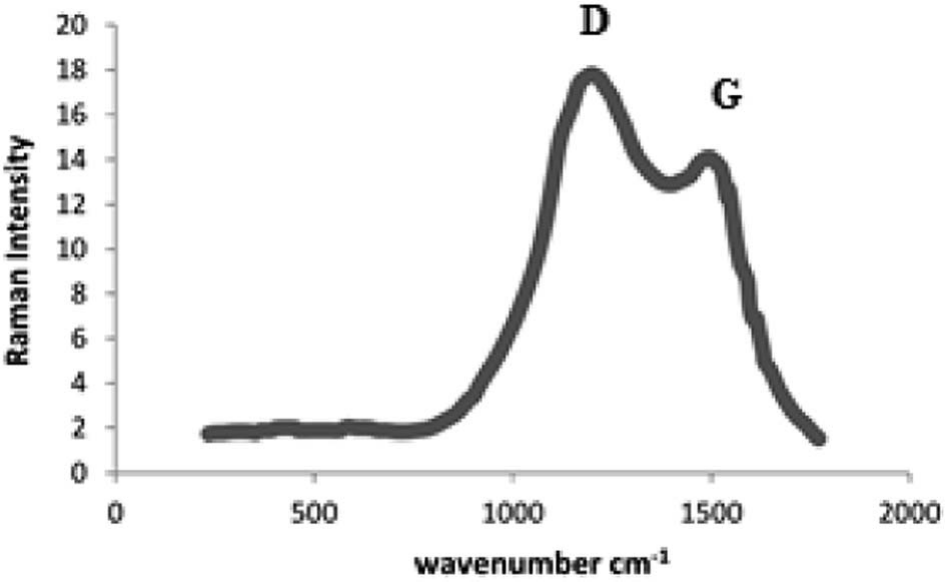
Raman spectrum of the as-synthesized N-rich porous carbon support.

Raman spectrum of the as-synthesized N-rich porous carbon support reveals two peaks at 1325 (D) and 1583 (G) cm^−1^ (Fig. 3). Peak D indicates the degree of disorder in the carbon structure, which emerged due to the presence of the heteroatom and blank spaces in the structure. These factors reduce the symmetry in the crystalline network of carbon and as a result create the disorder. Peak G is related to the graphitic carbon, and the relative intensities of peaks D and G characterize carbon graphitization. Raman spectroscopy analysis indicates the presence of nitrogen and the structural defects caused by nitrogen.

**Fig. 4:**
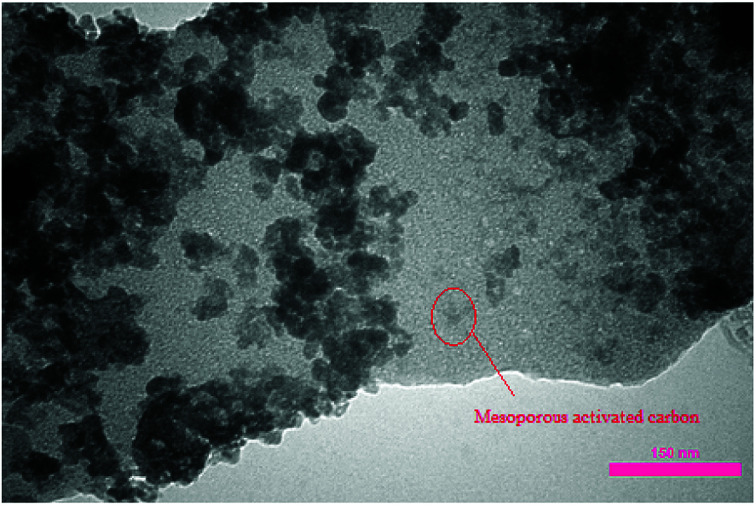
TEM analysis of NAC-Cu.

After the immobilization of copper NPs, transmission electron microscopy (TEM) analysis was performed for verifying the attachment of copper NPs on carbon nanosupport. As can be seen in Fig. 4, the NPs are successfully stabilized on the porous carbon bearing nanocavities. The size of Cu NPs was determined to be about 40–80 nm.

**Fig. 5:**
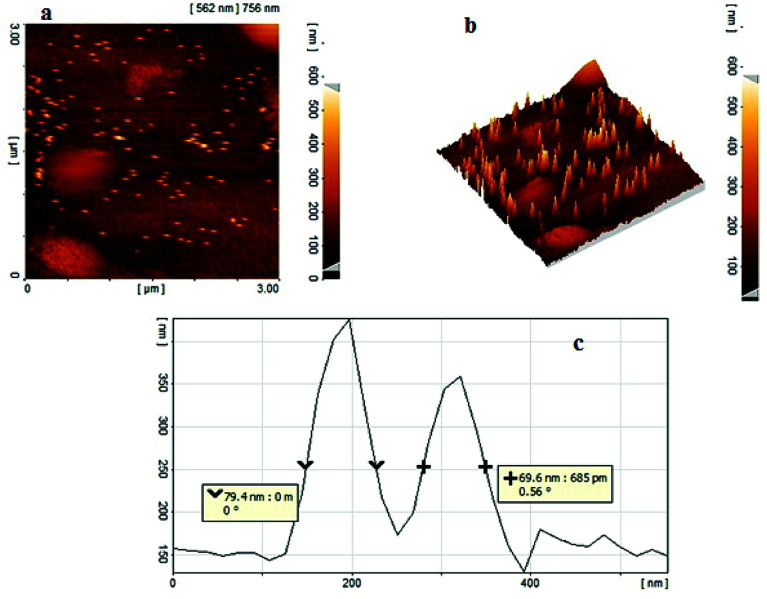
AFM images of NAC-Cu; (a) 2-D, (b) 3-D and (c) voltage profile.

The size and morphology of copper nanoparticles were also investigated by recording two-dimensional (2-D, Fig. 5a) and three-dimensional (3-D, Fig. 5b) AFM images. The AFM analysis of copper nanoparticles reveals spherical nanoparticles with a uniform distribution on NAC. The average size of the copper nanoparticles was estimated to be 50–80 nm according to the voltage profile of the AFM image (Fig. 5c).

**Fig. 6:**
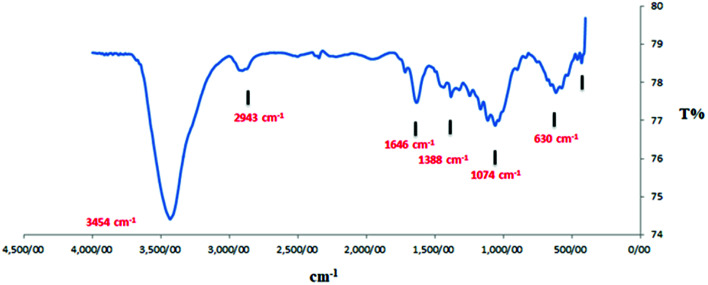
FT-IR spectrum of NAC-Cu.

The available functional groups of the as-synthesized nanosupport have been investigated using the FT-IR technique. The presence of the peaks at 1632–1381 cm^−1^, related to C=C and C=N bonds, confirmed the successful conversion of the biomass material to the carbon support as well as the presence of nitrogen in its structure (Fig. 6).

Next, we turned our attention to applying the NAC-Cu nanocatalyst for the reaction of phenylacetylene with a series of alkyl azides to obtain the corresponding 1,4-disubstituted 1,2,3-triazoles 3. To find the optimal conditions, the reaction of phenylacetylene, sodium azide and benzyl bromide was used as a model reaction. A mixture of phenylacetylene 1 (1 mmol), benzyl bromide 2a (1 mmol), sodium azide (1.2 mmol) and solvent (5 mL) was stirred under various reaction conditions (Table 1).

**Table 1:** NAC-Cu catalyzed the model reaction of phenylacetylene, sodium azide and benzyl bromide under various conditions^a^

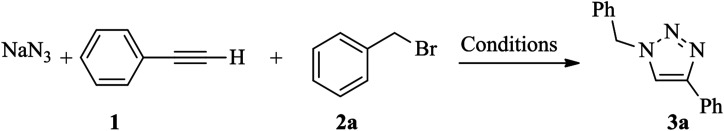				
Entry	Solvent	Catalyst^b^ (mol%)	Time (h)	Yields^c^ (%)
1	H_2_O	—	8	Trace
2	H_2_O	NAC (0.25 g)	8	Trace
3	H_2_O	NAC-Cu (2)	1.5	92
4	EtOH	NAC-Cu (2)	1.5	Trace
5	H_2_O/EtOH	NAC-Cu (2)	1.5	95
6	H_2_O	NAC-Cu (1)	1.5	80
7	H_2_O	NAC-Cu (0.5)	5	60
8	H_2_O	AC-Cu (2)^d^	24	50

a Reaction conditions: solvent (5 mL), phenylacetylene 1 (1 mmol), benzyl bromide 2a (1 mmol), sodium azide (1.2 mmol) at room temperature.

b Based on copper nanoparticles.

c Isolated yield.

d Copper supported on commercial activated carbon.

As shown in Table 1, in the absence of catalyst the product was not produced even after 8 h (entry 1). The catalytic activity of the N-rich carbon support was also studied under the same conditions, and no desired product 3a was obtained (entry 2). The click reaction with 2 mol% of the NAC-Cu in water resulted in the desired product 3a after 1.5 h with high yield (entry 3). Changing the solvent to ethanol led to the decrease in the reaction efficiency because sodium azide is not very soluble in ethanol (entry 4). In the mixture of water/ethanol, the yield of the reaction increased to 95% in 1.5 h (entry 5), indicating that the efficiency in water and water/ethanol mixture is nearly the same. For this reason, water was selected as the optimum solvent. Recently, a procedure for the synthesis of 1,2,3-triazole derivatives *via* a three-component coupling reaction in the presence of 1 mol% copper/carbon (Cu/C) catalyst was reported.^19^ Therefore, to reveal the effect of N-doped carbon in the reaction medium, the composite of Cu nanoparticles immobilized on commercial activated carbon (AC-Cu) was also evaluated for the model reaction (entry 8). Clearly, in the presence of NAC-Cu, the reaction time reduced by 16-fold and the yield increased compared with that of AC-Cu (92% *versus* 50%, Table 1, entries 3 and 8). By decreasing the amount of catalyst from 2 mol% to 0.5 mol%, the yield decreased to only 60% even after 5 h. As a result, the conditions of entry 3 were selected as the optimized reaction conditions. After achieving the optimized reaction conditions, we a series of the benzyl bromide derivatives were used as substrates to obtain the corresponding 1,2,3-triazoles (3). In general, electron withdrawing and releasing motifs furnished higher reaction rates, affording the desired products in excellent yields (Table 2). The structures of the products (3) were confirmed by CHN, ^1^H and ^13^C NMR, and FT-IR analyses and compared with those of the authentic samples previously reported.

**Table 2:** Efficient and green synthesis of 1,2,3-triazole derivatives catalyzed by NAC-Cu

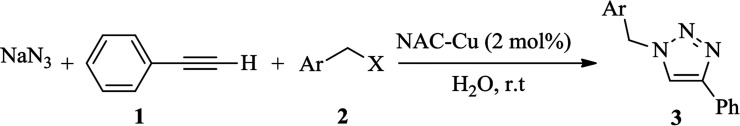				
Entry	Alkyl halide 2	Product 3	Time (min)	Yield^a^ (%)
1	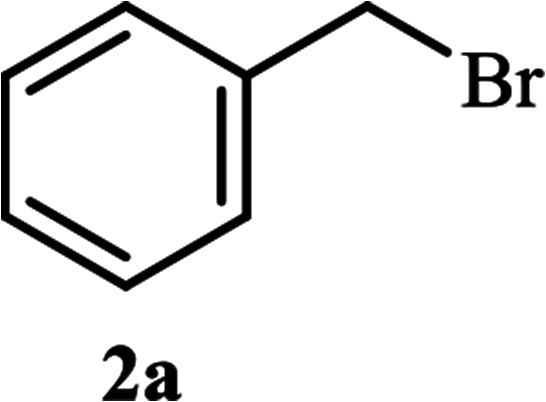	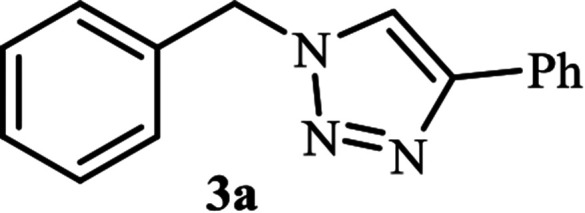	90	91
2	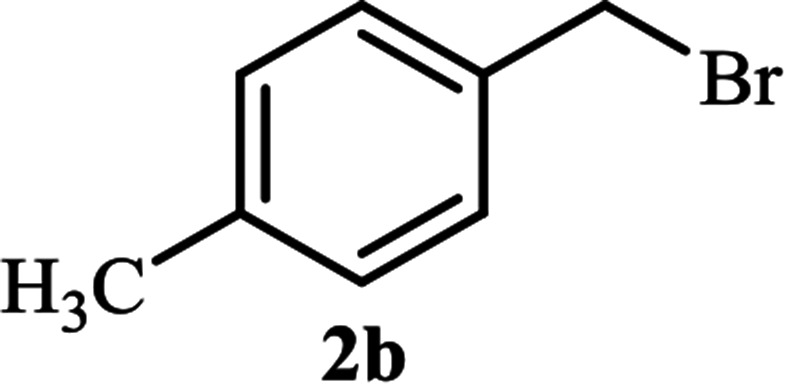	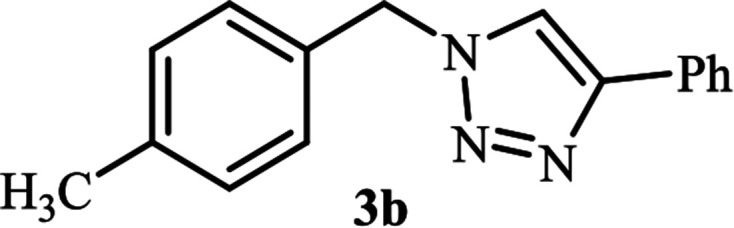	90	90
3	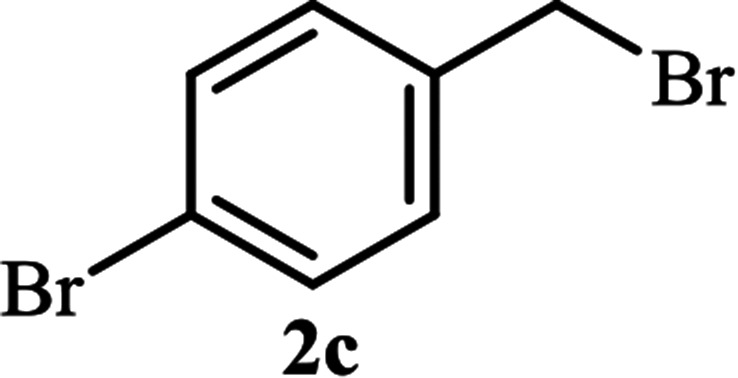	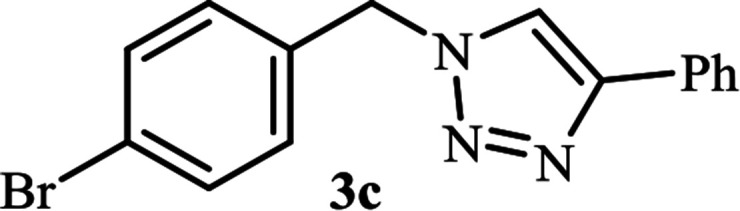	120	90
4	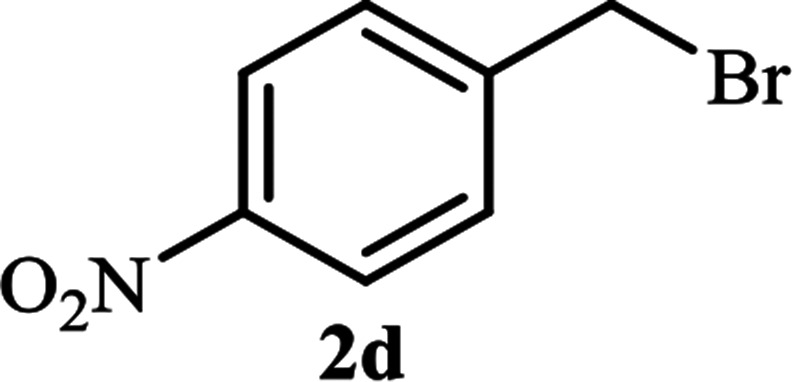	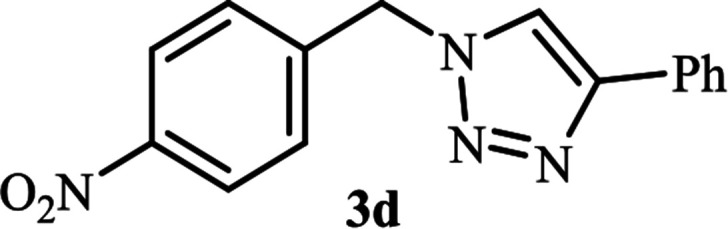	120	89
5	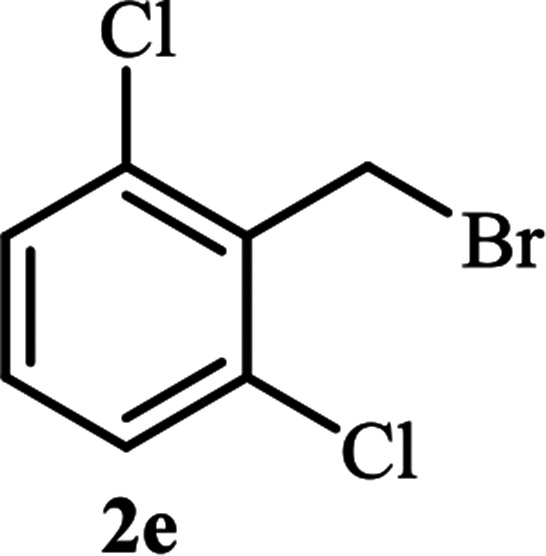	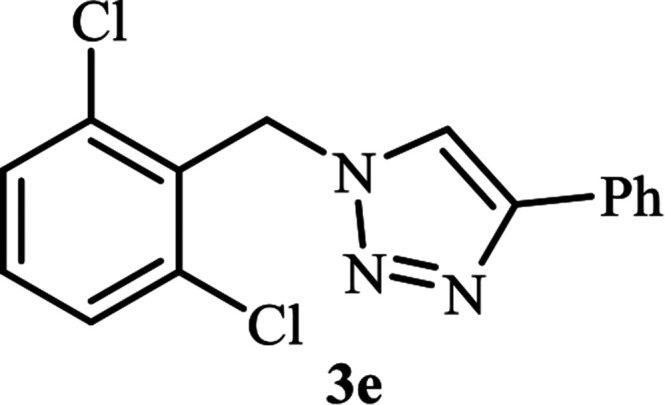	140	91

a Isolated yield.

**Fig. 7:**
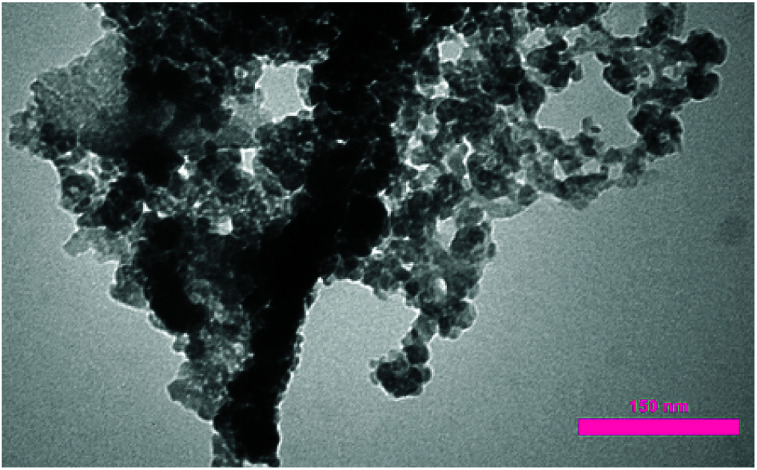
TEM analysis of recycled NAC-Cu.

The reusability of the NAC-Cu nanocatalyst was studied for the model reaction. The results indicated that the catalytic activity did not decrease even after recycling the catalyst eight times, and the recycled copper nanoparticles remained intact on the carbon structure, as confirmed by TEM analysis (Fig. 7).

## 4. Conclusion

The porous carbon support has been synthesized from the protein of egg white biomass for the first time. After immobilizing the copper nanoparticles on the carbon support by using a chemical reduction method, the catalyst was used for the synthesis of 1,2,3-triazole derivatives. The structure of the nanocatalyst was determined using elemental analysis, FT-IR, Raman, TEM, AFM, AAS, BET and BJH techniques. The results illustrated that the copper nanoparticles had been successfully loaded onto the nitrogen-rich carbon support with uniform distribution. The click reaction was studied using this catalyst. The catalyst showed good activity in water as a solvent during the reaction. Furthermore, the catalytic activity of the as-synthesized catalyst did not been decrease after recycling the catalyst eight times, indicating remarkable stability of the immobilized copper nanoparticles on the support.

## Conflicts of interest

There are no conflicts to declare.

## Supplementary Material
